# Verification of Death

**Published:** 1913-06-14

**Authors:** Jas. R. Williamson

**Affiliations:** 100 Chedington Road, Upper Edmonton, London, N.


### Verification of Death.

*To the Editor of* The Hospital.

Sir,—Referring to the interesting report of the York County Hospital Inquiry in your excellent journal of the 7th inst., may I mention that the Association for the Prevention of Premature Burial seeks by legislation to enforce careful and effective verification of death in all cases before a certificate of death is signed by a medical man, and to establish a system of scientifically equipped and managed waiting mortuaries, such as have with much advantage existed for many years in Germany and Austria? It is believed that by these means the possibility of premature burial, through apparent death being mistaken (as it often has been and is) for permanent cessation of life, may be obviated.

At a meeting of the Hexham Guardians some time ago, in reply to Mr. Henry Straker, Mr. Lowry, a Local Government inspector, who was present, said: "Only a short while ago I myself heard of a case in which a supposed corpse lay for three distinct days, and yet that person came back to life. It would have been a terribly serious thing if that 'corpse' had been in the workhouse, as the interment in all probability would have taken place before the three days had elapsed." He added that he "was thoroughly with Mr. Straker in the matter that the doctors should make an examination. They were paid for the work, and should see the body after death and certify to the best of their ability that the person had ceased to live." This obviously applied to medical officers of workhouses, but in general practice kindly allow me to say certificates of death are more often than not given by medical men who do not trouble themselves to ascertain personally that death has really occurred, and the supposed dead are frequently buried before the only trustworthy proof of death, putrefactive decomposition, has set in.—I am Sir, your obedient servant,

Jas. R. Williamson.

100 Chedington Road, Upper Edmonton,
London, N.
June 9, 1913.

